# The independence of expression and identity in face-processing: evidence from neuropsychological case studies

**DOI:** 10.3389/fpsyg.2015.00770

**Published:** 2015-06-09

**Authors:** Sarah Bate, Rachel Bennetts

**Affiliations:** Department of Psychology, Faculty of Science and Technology, Bournemouth University, Poole, UK

**Keywords:** face recognition, face-processing, emotional expression, facial identity, prosopagnosia

## Abstract

The processing of facial identity and facial expression have traditionally been seen as independent—a hypothesis that has largely been informed by a key double dissociation between neurological patients with a deficit in facial identity recognition but not facial expression recognition, and those with the reverse pattern of impairment. The independence hypothesis is also reflected in more recent anatomical models of face-processing, although these theories permit some interaction between the two processes. Given that much of the traditional patient-based evidence has been criticized, a review of more recent case reports that are accompanied by neuroimaging data is timely. Further, the performance of individuals with developmental face-processing deficits has recently been considered with regard to the independence debate. This paper reviews evidence from both acquired and developmental disorders, identifying methodological and theoretical strengths and caveats in these reports, and highlighting pertinent avenues for future research.

## Introduction

The human face conveys information regarding a person’s identity (e.g., Schyns and Oliva, 1999; Gosselin and Schyns, 2001), emotional state (e.g., Ekman and Friesen, 1976; Smith et al., 2005), gender (e.g., Brown and Perrett, 1993), age (e.g., George and Hole, 1995), and direction of attention (e.g., Sander et al., 2007). This information is rapidly filtered so complex perceptual categorizations can be performed (Schyns et al., 2009). Despite decades of research on how we extract various cues from faces, it is still widely debated whether identity and expression information is processed and represented within shared or independent systems.

One way to approach this question is to examine the type of basic visual information (e.g., spatial frequency ranges or spatial location of diagnostic cues) and perceptual processes (e.g., holistic and featural or analytic processing) that are used to make identity and expression judgments. Studies examining the effect of spatial frequency (i.e., coarse vs. fine visual information) do not show a strict dissociation between expression and identity judgments: although certain spatial frequency bands may be more conducive to identification or detection of individual emotions (e.g., Costen et al., 1996; Gao and Maurer, 2011; Kumar and Srinivasan, 2011), or may be used preferentially for different tasks (e.g., Schyns and Oliva, 1999; Deruelle and Fagot, 2005); both high and low spatial frequency bands carry sufficient information to convey expression and identity information (e.g., Deruelle and Fagot, 2005), and biases toward spatial frequency bands are not fixed (Schyns and Oliva, 1999). As such, spatial frequency bands cannot be taken to represent dissociable pathways of visual processing for expression and identity. However, techniques such as the “Bubbles” task (Gosselin and Schyns, 2001) indicate that typical perceivers do focus or rely on subtly different areas of the face when making expression and identity judgments—for instance, perceivers use a variety of discrete facial regions for different expression judgments (e.g., Smith et al., 2005), whereas identity judgments rely on a more diffuse area of the face, encompassing the eyes, nose, and mouth (Gosselin and Schyns, 2001; Schyns et al., 2002). Taken together, these findings are consistent with the idea that facial identity information is processed in a holistic manner (see Maurer et al., 2002; Piepers and Robbins, 2012; for a definition and discussion of holistic processing), encompassing both individual facial features and their precise spatial configuration; whereas expression judgments may rely more on processing of individual components or conjunctions of components. For example, research using the composite task has found that, in general, identity judgments rely quite strongly on the integration of information from the top and bottom halves of the face (see Rossion, 2013 for a review). Expression judgments also show this “composite effect,” suggesting that expressions are also processed holistically, but some authors have suggested that this process is independent of or different from the holistic processing that occurs for identity (Calder et al., 2000; White, 2000). Furthermore, other studies have suggested that componential or part-based processing is more efficient, and hence the default route of visual processing, for expression judgments (Tanaka et al., 2012; see also Ellison and Massaro, 1997). In sum, then, expression and identity judgments may rely on subtly different visual cues and processing styles, with identity judgments making use of more diffuse spatial areas of the face and a processing style that integrates information from across these areas, and expression judgments relying on smaller spatial areas (incorporating one or two facial features) and a more piecemeal or componential processing style. While this gives some indication that identity and expression may make use of similar, or at least overlapping, visual information, it still leaves open the question of whether individuals access these visual cues or processing styles separately for different tasks, or whether they are irrevocably intertwined. As such, this paper focuses on research into neuropsychological case studies, and the contribution they can make to this debate.

**FIGURE 1:**
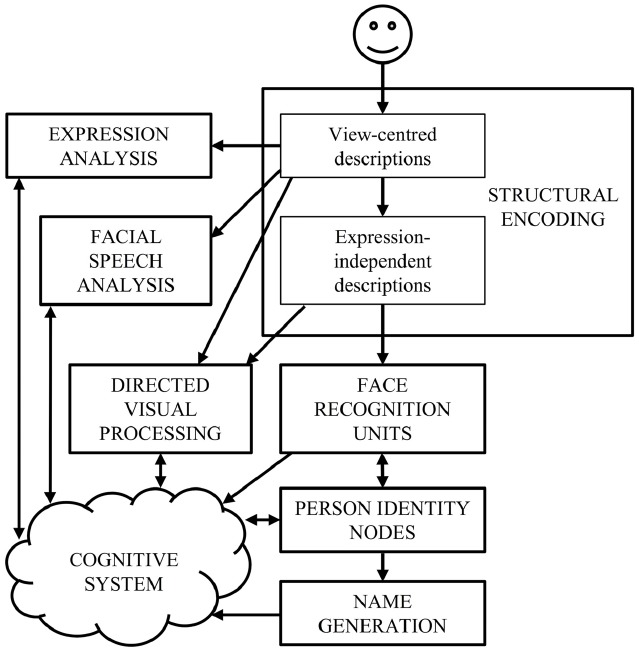
**The cognitive model of face-processing proposed by Bruce and Young (1986)**.

**FIGURE 2:**
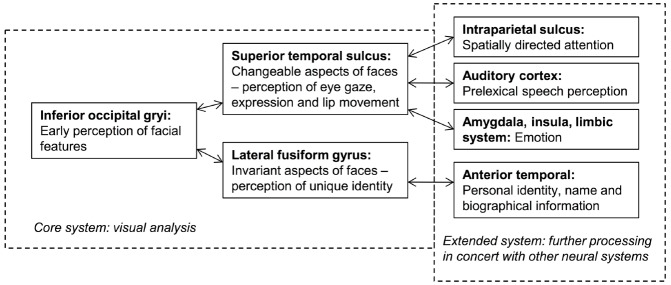
**An adaptation of the distributed model of face-processing proposed by Haxby et al. (2000)**.

Current face-processing models support the segregation of identity and expression mechanisms. Functional models posit that identity and expression information is processed independently, although some interaction may be mediated by the wider cognitive system (e.g., Bruce and Young, 1986; see Figure 1). Anatomical models (e.g., Haxby et al., 2000; Gobbini and Haxby, 2007) distinguish between static structural properties of the face that relate to a single person (e.g., the shape and spacing of the facial features provide information on facial identity: Sergent, 1985), and dynamic variant information that is common to many individuals (e.g., positions of the muscles that convey an emotional state). Anatomically distinct brain regions are believed to analyze this information, with the lateral fusiform gyrus processing identity and the superior temporal sulcus (STS) expression (see Figure 2). While the anatomical model predicts this split occurs after an early stage of perceptual processing in the inferior occipital gyrus, other authors suggest this phase involves higher-level processing of both expression and identity (Calder and Young, 2005; Palermo et al., 2013). Both accounts are broadly consistent with the patterns of visual information use outlined above.

Early evidence supporting the proposed independence of identity and expression processing (hereon termed “the independence hypothesis”) came from a double dissociation between two neurological disorders. One half comes from individuals with prosopagnosia, who cannot recognize familiar people yet have preserved processing of facial expression (e.g., Tranel et al., 1988). The other half comes from patients who are impaired at recognizing emotional expressions despite intact identification abilities (e.g., Young et al., 1993; Hornak et al., 1996). While the independence hypothesis remains a dominant aspect of cognitive and anatomical models, much behavioral (e.g., Fox and Barton, 2007; Bate et al., 2009a; Campbell and Burke, 2009) and neuroimaging (e.g., Ganel et al., 2005; Fox et al., 2009) evidence has questioned the degree of separation between the two processes. Further, Calder and Young (2005) discounted the traditional patient-based evidence supporting the independence hypothesis, positing that a single model can achieve independent coding of identity and expression using the different types of visual information described above.

Since the publication of Calder and Young’s (2005) review, new patient reports have overcome the limitations of previous work. These papers also describe neuroanatomical data that complement behavioral performance, and some directly assess the use of perceptual information in expression and identity processing. Further, several developmental neuropsychological case studies have addressed the independence hypothesis. This paper summarizes the patient-based evidence reported since 2005, and presents a timely review of the contribution of neuropsychological case reports to the independence debate.

## Acquired Deficits in Facial Identity Processing

One half of the neuropsychological double dissociation traditionally believed to support the independence hypothesis comes from individuals with prosopagnosia. This condition typically results from occipitotemporal lesions (Barton, 2008), and is characterized by a severe impairment in facial identity recognition. In some cases expression recognition appears to be preserved (i.e., performance was within the range of typical age-matched controls; Tranel et al., 1988; McNeil and Warrington, 1993; Young et al., 1993; Takahashi et al., 1995), supporting the independence hypothesis. Yet, Calder and Young (2005) argue that the bulk of this evidence should be discounted because the prosopagnosia is not visuoperceptual in origin, instead resulting from prosopamnesia (impaired recognition of faces encoded after but not before illness onset; e.g., Tranel et al., 1988), general amnesia, or more general semantic impairments (e.g., Etcoff, 1984). In fact, the authors suggested that only two cases truly appeared to have visuoperceptual deficits (Bruyer et al., 1983; Tranel et al., 1988), yet both investigations suffered from methodological or statistical limitations. The authors therefore concluded that no case of prosopagnosia published prior to their review provided convincing evidence in support of the independence hypothesis.

However, since 2005, further instances of prosopagnosia with preserved expression recognition have been described. Riddoch et al. (2008) reported patient FB, who had damage to the right fusiform, right inferior temporal, right middle temporal and right inferior occipital gyri. FB had severe prosopagnosia yet scored within the typical range on an expression recognition test. Yet, the actual processing strategies used by FB were not assessed, and, in line with previous patient reports (e.g., Adolphs et al., 2005), the authors suggest that her normal performance on the expression task may be underpinned by atypical strategies. As mentioned above, expression processing may rely on both part-based analytical processing and some degree of holistic processing. While the possibility was not explicitly tested, the authors suggest that FB relied on part-based information to a greater extent than controls in the expression task, and could use this information to achieve a normal score (see also Baudouin and Humphreys, 2006).

Fox et al. (2011) used more sensitive measures of identity and expression perception in four individuals with prosopagnosia, alongside a fMRI-based functional localizer that identified preserved and impaired cortical regions. All four patients had selective difficulties in facial identity recognition, and, consistent with the anatomical model (Haxby et al., 2000), two had right inferotemporal lesions and two had damage within the anterior temporal lobes. Strikingly, the authors also described a fifth patient with selective damage to the posterior STS (pSTS), who presented with impaired expression recognition. Notably, however, these deficits extended to identity recognition when irrelevant variations in expression needed to be discounted. While evidence from the four prosopagnosic individuals supports the independence hypothesis, the latter participant suggests some overlap in the diagnostic facial information used in the two tasks, although exactly what diagnostic information (e.g., processing style; separate visual cues) is currently unclear.

Converging evidence comes from patient HJA, who acquired damage to the ventral occipital and temporal lobes and was unable to recognize facial identity or static facial expressions at normal levels (Humphreys et al., 1993), potentially due to abnormal use of visual information during static expression processing (Baudouin and Humphreys, 2006). However, HJA performed significantly better when identifying moving facial expressions (Humphreys et al., 1993) and when matching moving faces based on identity (Lander et al., 2004). Although this movement advantage did not extend to overt identification or face learning, HJA’s use of movement cues in both identity and expression decisions suggests that (a) the neural mechanisms subserving facial movement processing—most likely the pSTS (Pitcher et al., 2011, 2014; Schultz et al., 2013)—can facilitate both processes, and (b) these neural mechanisms can be dissociated from those involved in static face-processing. This latter hypothesis is encompassed within the anatomical model (see Figure 2), although the role of the pSTS in identity recognition is less clear. O’Toole et al. (2002) suggest that the structure may also process “dynamic facial signatures” (characteristic patterns of motion that aid identification), although facial movement may also boost identity recognition by attracting attention and foveal fixation that guides stimulus-driven selective attention (O’Toole et al., 2002; Abrams and Christ, 2003). Regardless, this information may contribute to both expression and identity judgments, yet a smaller influence on the latter may facilitate identification only under limited circumstances (e.g., when the individual has trouble processing static facial cues; see O’Toole et al., 2002, for a discussion).

While increasing evidence supports independent mechanisms for dynamic and static facial information, there is less agreement about the stage at which this split occurs. Haxby et al. (2000) concur with Bruce and Young (1986) that the bifurcation occurs at an early stage (i.e., before the formation of view-independent structural descriptions in the functional model, and before processing in the lateral fusiform gyrus for identity and the STS for expression in the anatomical model). However, Palermo et al. (2011, 2013) posit a higher-order shared stage of processing that may involve holistic processing. A very recent report supports this hypothesis. Richoz et al. (2015) investigated the decoding of facial expression in patient PS, who had major lesions in the left mid-ventral and the right inferior occipital cortex, and minor lesions of the left posterior cerebellum and the right middle temporal gyrus. Previous work by Caldara et al. (2005) indicated that, in a facial identity task, PS used information in a sub-optimal manner, focusing on the mouth and external contours and avoiding the eye region. Yet, Richoz et al. (2015) found that PS used all the facial features to decode dynamic emotional expressions (with the exception of fear), and performed within the typical range when classifying those expressions. Despite this, PS had a general impairment in categorizing many static facial expressions, which, in line with the theory of Palermo et al. (2013), the authors attribute to the right inferior occipital gyrus lesion. They suggest that the preserved processing of dynamic expressions may occur via a direct and functionally distinct pathway connecting early visual cortex to the pSTS. Although this study did not investigate whether the patient also benefited from dynamic information in identity judgments, it indicates that the use of both dynamic and static information should be assessed in expression and identity tasks. Indeed, dissociable anatomic systems may exist for dynamic and static information, but there may be some overlap in the diagnostic information used for expression and identity judgments in each pathway.

## Acquired Deficits in Facial Expression Recognition

There are fewer reports of acquired deficits in expression processing and it is not always clear whether facial identity recognition has been preserved. There is also some variation in lesion location (which is sometimes under-specified), with early studies reporting expression recognition difficulties following diffuse bilateral damage (Kurucz et al., 1980), right (Adolphs et al., 1996) or left (Young et al., 1993) hemisphere lesions, or selective amygdala damage (Adolphs et al., 1994; Brierley et al., 2004). These reports may be reconciled by findings that perceptual and recognition processes may be independently affected, and this may be related to lesion location. Studies have demonstrated deficits in expression matching but not naming following right hemisphere damage (Young et al., 1993), particularly to the right pSTS (Fox et al., 2011, patient R-ST1). Conversely, deficits in expression naming and emotional memory have been reported following unilateral (Adolphs et al., 1999; Brierley et al., 2004; Fox et al., 2011, patient R-AT1) or bilateral (Brierley et al., 2004) amygdala damage, respectively. Patient R-AT1 in Fox et al.’s (2011) report showed no pSTS damage and no difficulty performing expression matching tasks, suggesting dissociable roles for the pSTS and amygdala in expression processing. These findings converge neatly with the anatomical model.

The studies reviewed above mostly relied on categorization performance rather than examination of processing strategy. One exception is Adolphs et al.’s (1994) report of patient SM, who presented with amygdala damage and a selective deficit in fear recognition. In a later report, the authors found that she was unable to use diagnostic information from the eye region, irrespective of emotional expression (Adolphs et al., 2005). This had presented as a selective impairment in fear recognition because the eyes are the most important feature for identifying this emotion. As noted above for investigations examining individuals with prosopagnosia, categorization performance alone may obscure atypical use of visual information, influencing the theoretical conclusions that can be gleaned from patient reports.

Other criticisms suggest this half of the double dissociation has been over-simplified (Calder and Young, 2005). Evidence suggests that a single processing stream cannot process all expressions, as dissociable neural systems have been identified for particular emotions (e.g., Krolak-Salmon et al., 2003; although it is not clear if these dissociations are based on the emotion itself or simply perceptual features that are embedded within that expression, see Whalen et al., 2004). Instead, selective disruption of expression recognition may reflect damage to a more general emotion system (Calder and Young, 2005). Indeed, facial expression impairments are often associated with deficits in decoding vocal expression for specific emotions (Sprengelmeyer et al., 1999; Calder et al., 2000, 2001) and at a more general level (Adolphs et al., 2002).

## Developmental Cases

Calder and Young (2005) rejected some patient-based evidence supporting the independence hypothesis on the grounds that it was developmental in origin. In line with other authors (e.g., Bishop, 1997; Karmiloff-Smith et al., 2003), they argue that individuals with developmental disorders may have had some form of atypical brain organization from birth. Thus, it is difficult to interpret developmental cases within cognitive models of the face-processing system, given that (a) the basic architecture of this system might not have developed, and (b) it is unknown if the system can be selectively disrupted in the same manner inferred for patients with acquired deficits. One could therefore argue that developmental rather than representational abnormalities provide a convincing explanation of the basis of face-processing impairments in these cases.

Despite these issues, several studies have investigated the independence hypothesis in developmental prosopagnosia. This disorder is typically viewed as a parallel condition to acquired prosopagnosia, and is similarly characterized by a severe and relatively selective deficit in facial identity recognition (e.g., Duchaine et al., 2007; Bate et al., 2009b; Garrido et al., 2009; Bate and Cook, 2012). However, these individuals have never experienced a brain injury, and do not have concurrent socio-emotional, intellectual or low-level perceptual difficulties. While some individuals with developmental prosopagnosia have deficits in facial expression recognition (Duchaine et al., 2006; Minnebusch et al., 2007), others appear to have a selective deficit only affecting the recognition of facial identity (Duchaine et al., 2003; Humphreys et al., 2007). Notably, however, Palermo et al. (2011) reported 12 developmental prosopagnosics who displayed normal levels of accuracy on a series of facial expression recognition tests, yet presented with impaired holistic coding of both facial expression and facial identity. The authors interpreted this finding as evidence that the prosopagnosics were relying on atypical strategies to achieve normal performance on the expression tasks. Thus, further work that examines actual processing strategy rather than accuracy and response times alone is required to clarify whether facial expression recognition is truly unaffected in some individuals with developmental prosopagnosia.

Evidence of selectively impaired expression recognition has been reported in socio-developmental disorders (SDDs), but findings are mixed. Many studies fail to find group-level deficits, with individuals falling into both typical and atypical ranges (for reviews, see Harms et al., 2010; Uljarevic and Hamilton, 2012). There is nevertheless some evidence that expression and identity processing can be dissociated (e.g., Hefter et al., 2005), although particular emotions (e.g., fear and surprise: Ashwin et al., 2007; Humphreys et al., 2007) appear to be disproportionately affected. As noted for brain-damaged patients, this evidence again suggests that deficits in expression recognition may be related to emotional processing more than face-processing. For instance, recent work suggests that facial (Cook et al., 2013) and vocal (Heaton et al., 2012) affect recognition deficits in autism may be attributed to co-occurring alexithymia rather than autism itself. Case reports of individuals with developmental visuoperceptual deficits in expression recognition without a concurrent SDD would provide more convincing evidence to support the independence hypothesis, yet no known case has been reported to date. This is unsurprising given that several authors suggest that facial expression recognition impairments inevitably lead to deficits in socio-emotional functioning, which may in turn lead to impairments in facial identity recognition (e.g., Schultz, 2005).

More consistent findings suggesting atypical processing of facial expression have been observed in studies that examine the use of diagnostic facial information in SDDs. Several reports suggest that, during expression recognition, high-functioning individuals with autism look less at the eye region (e.g., Pelphrey et al., 2002; Corden et al., 2008) or the inner facial features (eyes, nose, and mouth; Hernandez et al., 2009; Bal et al., 2010) than controls, or do not effectively use information from the upper-face when decoding expressions (Spezio et al., 2007; Gross, 2008).

## Conclusion

While patient-based evidence supporting the independence of facial identity and expression processing was almost entirely discounted by Calder and Young (2005), more recent neuropsychological case studies have provided more convincing evidence supporting the independence hypothesis, particularly when accompanied by neuroanatomical data. Yet, reliance on recognition performance alone can clearly obscure atypicalities in the use of diagnostic visual information in both identity and expression recognition; and the use of dynamic and static information should be assessed in both processes, at both perceptual and mnemonic levels. When tested appropriately, neurological patients can provide invaluable contributions to theoretical debates such as the independence hypothesis. Although suitable patients are rare, supporting evidence may also be gleaned from more readily available developmental cases. While some authors have discounted the contribution of these individuals on theoretical grounds, observation of similar patterns of performance across acquired and developmental disorders would ultimately provide more convincing insights into the independence debate.

### Conflict of Interest Statement

The authors declare that the research was conducted in the absence of any commercial or financial relationships that could be construed as a potential conflict of interest.
